# Prem Baby Triple P: a randomised controlled trial of enhanced parenting capacity to improve developmental outcomes in preterm infants

**DOI:** 10.1186/s12887-015-0331-x

**Published:** 2015-03-04

**Authors:** Paul Colditz, Matthew R Sanders, Roslyn Boyd, Margo Pritchard, Peter Gray, Michael J O’Callaghan, Virginia Slaughter, Koa Whittingham, Peter O’Rourke, Leanne Winter, Tracey Evans, Michael Herd, Jessica Ahern, Luke Jardine

**Affiliations:** 1 The University of Queensland Centre for Clinical Research, Faculty of Health Sciences, The University of Queensland, Royal Brisbane and Women’s Hospital, Brisbane, Australia; 2 The Parenting and Family Support Centre, School of Psychology, Faculty of Social and Behavioural Sciences, The University of Queensland, Brisbane, Australia; 3 Royal Brisbane and Women’s Hospital, Brisbane, Australia; 4 Queensland Cerebral Palsy and Rehabilitation Research Centre, School of Medicine, Faculty of Health Sciences, The University of Queensland, Brisbane, Australia; 5 Mater Mothers’ Hospital, Brisbane, Australia; 6 Early Cognitive Development Centre, The School of Psychology, Faculty of Social and Behavioural Sciences, The University of Queensland, Brisbane, Australia; 7 QIMR Berghofer Medical Research Institute, Brisbane, Australia; 8 The School of Psychology, The University of Queensland, Faculty of Social and Behavioural Sciences, The University of Queensland, Brisbane, Australia

**Keywords:** Preterm infants, Behavioral family intervention, Child behavioral adjustment, Child emotional adjustment, Child cognitive and language development, Parenting support/education

## Abstract

**Background:**

Very preterm birth (<32 weeks gestation) is associated with motor, cognitive, behavioural and educational problems in children and maternal depression and withdrawal. Early interventions that target parenting have the greatest potential to create sustained effects on child development and parental psychopathology. Triple P (Positive Parenting Program) has shown positive effects on child behaviour and adjustment, parenting practices and family functioning. Baby Triple P for Preterm infants, has been developed to target parents of very preterm infants. This study tests the effectiveness of Baby Triple P for Preterm infants in improving child and parent/couple outcomes at 24 months corrected age (CA).

**Methods/Design:**

Families will be randomised to receive either Baby Triple P for Preterm infants or Care as Usual (CAU). Baby Triple P for Preterm infants involves 4 × 2 hr group sessions at the hospital plus 4 × 30 min telephone consultations soon after transfer (42 weeks C.A.). After discharge participants will be linked to community based Triple P and intervention maintenance up to 24 months C.A. Assessments will be: baseline, post-intervention (6 weeks C.A.), at 12 and 24 months C.A. The primary outcome measure is the Infant Toddler Social & Emotional Assessment (ITSEA) at 24 months C.A. Child behavioural and emotional problems will be coded using the mother-toddler version of the Family Observation Schedule at 24 months C.A. Secondary outcome will be the Bayley Scales of Infant and Toddler Development (BSID III) cognitive development, language and motor abilities. Proximal targets of parenting style, parental self-efficacy, parental mental health, parental adjustment, parent-infant attachment, couple relationship satisfaction and couple communication will also be assessed. Our sample size based on the ITSEA, has 80% power, predicted effect size of 0.33 and an 85% retention rate, requires 165 families are required in each group (total sample of 330 families).

**Discussion:**

This protocol presents the study design, methods and intervention to be analysed in a randomised trial of Baby Triple P for Preterm infants compared to Care as Usual (CAU) for families of very preterm infants. Publications of all outcomes will be published in peer reviewed journals according to CONSORT guidelines.

**Trial registration:**

Australian New Zealand Clinical Trials Registry: ACTRN12612000194864.

## Background

### Ensuring a healthy start to life for very preterm babies

Approximately 1.5% of babies are born very preterm at <32 weeks gestation, equating to 2899 babies p.a. admitted to an Australian neonatal intensive care nursery [1]. Most admitted to neonatal intensive care survive (≈85%), but 10% develop major disabilities such as cerebral palsy and 50% develop intellectual, educational and/or behavioural problems [2]. These problems cause emotional and financial stress for families and society.

### Disability in very preterm babies

Children born very preterm are at increased risk of behavioural and emotional problems at 2 years corrected age (C.A.) including internalising and dysregulation difficulties as measured by the Infant Toddler Social & Emotional Assessment (ITSEA) [3]. Clinically relevant, pervasive behaviour problems are 2–9 times more common in preterm than in term born infants [4]. Behavioural difficulties early in childhood have implications for the developmental trajectory including schooling, social development and mental health. Children born very preterm experience problems across educational domains and as a result, approximately 40% require special educational assistance and 20% repeat a grade in primary school. Attentional problems, poor postural control and hyperactivity are prevalent; Attention Deficit-Hyperactivity Disorder is 3–6 times more common [5]. Mean general intelligence (IQ) score is about ^2^/_3_ SD (i.e. 10 points) below that of term born peers [6]. The learning and behavioural impairments in children born very preterm are associated with numerous ‘medical’ risk factors such as gestational age, periventricular haemorrhage, periventricular leucomalacia, respiratory distress syndrome, necrotising enterocolitis, suboptimal nutrition/growth and therapeutic exposures, but collectively these factors account for only a portion of the variance associated with long-term outcomes [6,7]. Social and environmental factors such as social class, parental education, parental mental health, parenting style, family structure, family functioning and the home environment also have major impacts on the development of children born very preterm [8,9]. Furthermore, families of children born very preterm are more likely to experience socioeconomic disadvantage [10] and this social risk is in turn associated with increased behavioural problems [3]. Infants born preterm are at high risk of a ‘double whammy’ of adversity, the initial being the biological adversity that preterm birth confers, and the subsequent being environmental adversity. This project focuses on optimising the developmental environment for the first 2 years through the pervasive neurodevelopmental influence of parenting.

### Cochrane reviews show that early interventions improve preterm outcomes

Early interventions have the potential to improve outcomes for children born very preterm but few babies receive high quality intervention due to the high costs (e.g. home visiting). Systematic reviews of existing early interventions [11,12] suggest that beneficial effects are present including improvements in cognitive outcomes in infancy (standard mean difference [SMD] 0.46 SD; 95% CI 0.36-0.57; *p* < 0.0001), and at preschool age (intelligence quotient SMD 0.46 SD; 95%CI 0.33-0.59; *p* < 0.0001) [12]. These improvements were not however sustained at school age [12]. Our Cochrane review concluded that further high quality RCTs with long term follow-up are needed to identify the potential of early developmental interventions in very preterm infants to produce sustained effects on cognitive, behavioural, motor and family outcomes [12,13]. A recently conducted RCT of a distributed model of developmental care at home demonstrated effects on maternal mental health and child externalising behaviour but with no effects on cognitive or motor outcomes [14]. The lack of sustained treatment effects for existing interventions suggests that a novel intervention that specifically addresses sustainability of effect may be beneficial. An intervention that focuses on sustained environmental enrichment through enhanced parenting practices is a promising approach. A key strength of the current study is that the parenting intervention is integrated into a successful, existing, funded community-based parenting program (Triple P) which will facilitate sustained exposure to the intervention at relatively low cost.

### Early interventions that target parenting hold the greatest potential

Of the early interventions that may impact child development, interventions that target parenting hold the greatest potential [15]. Parenting interventions have the potential to create sustained effects on child development at a relatively low cost as changes in the family system continue to support changes in the child’s developmental trajectory over time. Several studies have confirmed that the quality of daily parent–child interactions powerfully impacts many domains of development throughout childhood [16,17]. Evidence from behavioural genetics, epidemiological, correlational and experimental studies demonstrates that parenting practices have a major influence on children’s development [18] including upon behavioural and emotional development [19], early language and social development [20] later executive processing skills [21] and academic achievement [17]. The influence of parenting practices on development has been confirmed in preterm infants, with changes in parental behaviour producing equal or greater effects on development in infants born preterm [20,22]. Parenting interventions, derived from social-learning, functional analysis, and cognitive-behavioural principles, are among the most powerful interventions available and are the treatment of choice for a number of developmental problems in toddlers and preschool aged children [23,24].

### Parents of infants born very preterm are at risk for parenting difficulties

Preterm birth is associated with maternal depression, withdrawal and low levels of maternal coordination with the infant [25]. These less-than-optimal features of early parent-infant interaction may contribute to the poor socio-emotional, behavioural, cognitive and language outcomes common in infants born preterm. Interventions to improve parental mental health may therefore impact on child outcomes. An RCT of a preventive care at home intervention for very preterm infants demonstrated significant differences in maternal depression (mean difference [MD] -2.0 95% CI −3.2 to −0.7; *p* = 0.003) and anxiety (MD −3.1 CI −4.5 to −1.6; *p* < 0.001) along with significant reductions in child externalizing (MD −4.1 CI −8.2 to −0.02; *p* = 0.05) and deregulation behaviours (MD −8.7 CI −13.2 to −4.2; *p* < 0.001) and improvements in child competence (MD 6.7 CI 0.7 to 11.8; *p* = 0.03) [14]. Further, in comparison to children of mothers who experienced a positive transition to parenthood, children of mothers who experienced postpartum adjustment difficulties have poorer cognitive development, including problem solving and visuomotor performance at age 1 and 4 years [26]. Infants born very preterm may also be at increased risk of abuse. Reported rates of referrals of 14.8% for child abuse and of substantiated cases at 8.8% are high [27]. Furthermore, parents of preterm infants themselves identify a need for support in their transition to parenting and a need for more information on how they can support their infant’s development [28].

### Triple P is a highly effective parenting intervention

Triple P (Positive Parenting Program) has been developed and evaluated over the past 30 years [15,29]. It is one of the most extensively evaluated and effective models of parenting intervention, and is now implemented in Australia and 25 other countries [30]. Triple P is a comprehensive population-level system of parenting and family support that incorporates a multi-level system of interventions targeting parents of children from infancy to adolescence. The aim of Triple P is to impact upon child outcomes at a population level through enhancing parenting practices. Various levels of the Triple P system have been subjected to controlled evaluations and consistently shown positive effects on observed and parent-reported child behaviour and adjustment, parenting practices, and parental adjustment [15]. The benefits of Triple P are not restricted to children and include beneficial effects on family functioning, including reduced maternal depression and stress, increased parental satisfaction and efficacy, and reduced couple conflict over parenting issues [31-34]. A 5-year RCT reported that Triple P significantly reduced population indicators of child maltreatment at a population level with effect sizes ranging from large to very large (d = 1.09 – d = 1.22) [35]. At least two independent meta-analyses drawing on 55 evaluation studies from different countries, research teams and child age groups have established the efficacy of Triple P in improving children’s behaviour and adjustment over and above improving parenting skills [36,37] and the efficacy of Triple P has been noted in a systematic review of all preventative interventions for child behavioural and emotional problems [38]. Effect sizes of the intervention on child outcome measures range from small-moderate (mean d = 0.4) with universal, low-risk populations to moderate-large for high risk and clinical populations (mean d = 0.7) [36]. There is also strong evidence that improvements in the domains of parenting and child behaviour have a positive effect on cognitive and school performance [15,26,37,39].

### Triple P specifically targeted to preterm infants – preliminary evaluation

Recently Baby Triple P, a tailored variant of Triple P for expectant couples of term born infants has been developed [40]. The development of Baby Triple P included extensive independent review of the research on risk and protective factors for adverse developmental outcomes in infancy as well as policy documents and UNICEF/WHO guidelines on infant care. An RCT of Baby Triple P in expectant couples (n = 128) examined the impact on infant problem behaviours, maternal depression and couple relationship satisfaction [40]. Couples receiving Baby Triple P reported high satisfaction with the intervention [40].

Recently a modified variant of Baby Triple P specifically targeting very preterm infants, Baby Triple P for Preterm infants has been developed for the present clinical trial (ACTRN1261200194864). The modification process was informed by qualitative research with parents of very preterm infants and involved the collection of extensive consumer preference data both qualitatively through focus groups and quantitatively through a national survey [41]. A focus group of parents of very preterm infants (n = 15) after viewing Prem Baby Triple P materials agreed that Baby Triple P for Preterm infants is appropriate, feasible and needed [42]. Parents identified numerous strengths in the intervention including (i) normalisation of preterm parenting, (ii) information about development, (iii) creating a safe environment, (iv) building a positive parent-infant relationship, (v) strategies to manage behavioural issues, (vi) building adaptive coping skills and (vii) a focus on ‘learning to parent together’. In a nation-wide survey (n = 123 parents of preterm infants; n = 32 parents of term infants) parents rated the program highly, with no significant differences between term, preterm and very preterm parent responses, *F*(8, 98) = 1.34, *p* >0.23 [41]. Parental perceptions of risk of poor child health and developmental delay predicted higher acceptability ratings in parents of very preterm infants *sr*^*2*^*.08*, *β* = −.30, *t* [58] = −2.34, *p* < 0.03, indicating that the material has been well adapted to preterm specific needs [41]. Baby Triple P for Preterm infants, that commences in the neonatal period in the neonatal unit and continues into an existing community-based parenting resource after hospital discharge, is expected to be straightforward to implement and is likely to be sustainable and efficacious because of the existing Triple P community resource.

### Baby Triple P for Preterm infants – the conceptual model

**Figure 1:**
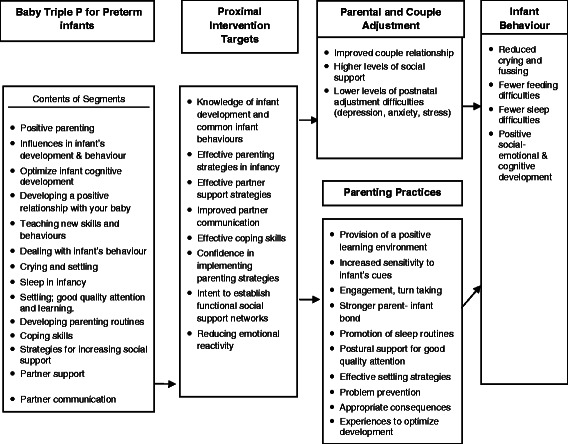
**Conceptual model of Baby Triple P for Preterm infants.**

Baby Triple P for Preterm infants has a strong conceptual basis with potential to make a positive contribution to family functioning and infant behavioural, cognitive and language development (Figure 1).

### Baby Triple P for Preterm infants key components

Baby Triple P for Preterm infants incorporates the key elements of teaching (i) effective parenting strategies, (ii) coping skills and (iii) partner support strategies.

- *Parenting strategies:* Numerous studies have shown a strong link between teaching parents effective parenting strategies based on behavioural principles and positive child developmental outcomes [43]. Interventions during infancy which focus on enhancing parenting self-efficacy [44], psychoeducation about infant development [20], sleep training & settling routines, and improving the parent–child attachment relationship [20,45] have positive effects on various measures of infant and parent outcomes.
- *Coping skills:* Teaching parents adaptive coping skills such as relaxation, cognitive strategies [32] (e.g. coping statements) and strategies to enhance the social support network [46,47], lead to positive outcomes for children and family functioning as a whole.
- *Partner Support:* Interventions that combine parenting education with a marital intervention component significantly improve outcomes for parents and children compared with parenting education alone [43,48]. For single mothers this component can be adapted to focus on support from a nominated significant other (e.g. the child’s grandparent).

### Broad aim of proposed study

To conduct a pragmatic RCT to determine whether Baby Triple P for Preterm infants compared to Care as Usual (CAU) optimises child outcomes including behavioural and emotional adjustment, cognitive and language development at 24 months C.A. in infants born very preterm (<32 weeks). In addition, the effect of Baby Triple P for Preterm infants on parenting self-efficacy, parenting style, parental mental health, mother-infant attachment, relationship satisfaction and couple communication at either post-intervention (6 weeks C.A.), at 12 months C.A. and 24 months C.A. compared to Care as Usual (CAU) will be investigated.

## Methods

**Figure 2:**
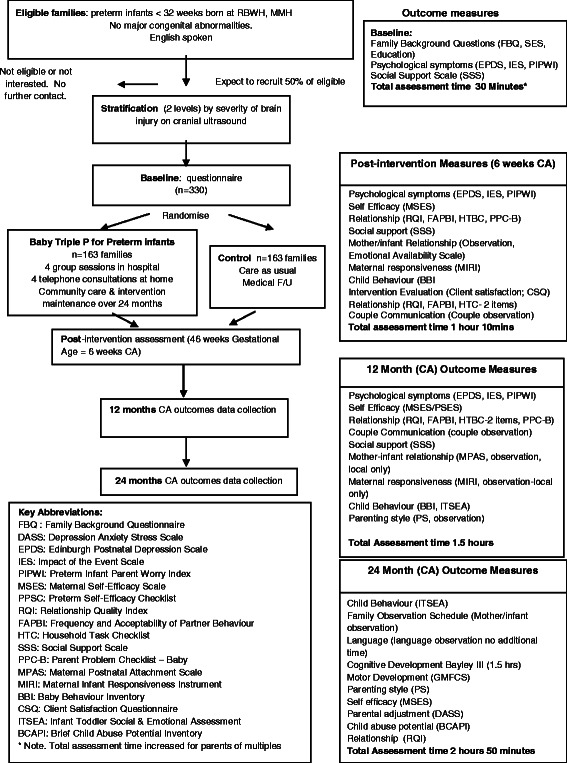
**Consort flow chart for Baby Triple P for preterm infants.**

An RCT will be conducted to evaluate whether Baby Triple P for Preterm infants compared to Care as Usual (CAU) optimises child and parent/couple outcomes at 24 months C.A. The flow chart of the study according to CONSORT guidelines is reported in Figure 2.

The primary hypotheses to be tested are:

- H^1^ Children whose parent/s participated in Baby Triple P for Preterm infants will score significantly higher on measures of behavioural and emotional adjustment at 24 months C.A. than children whose parents received CAU.
- H^2^ Children whose parent/s participated in Baby Triple P for Preterm infants will score significantly higher on measures of cognitive, language and motor development (BSID III) than children whose parents received CAU at 24 months C.A.

The secondary hypotheses to be tested are:

- H^3^ Parents who participated in Baby Triple P for Preterm infants will have significantly better parent and couple outcomes than parents who received CAU (including parenting style, parental self-efficacy, parental mental health and relationship satisfaction). Whether these proximal factors are important in mediating the treatment effect on primary and secondary child outcomes at 24 months C.A. will be tested.

### Study sample and recruitment

Participants will be preterm infants (born <32 weeks’ gestation) and their families admitted to the Neonatal Intensive Care Units (NICUs) at the Royal Brisbane and Women’s Hospital (RBWH) and Mater Mothers’ Hospital Brisbane (MMH).

### Inclusion criteria

1. The infant must have a gestational age at birth of less than 32 weeks.
2. The infant’s parents must agree to the assessment requirements of the study.

### Exclusion criteria

The study will exclude infants and their parents in the case of:

1. The infant having major congenital anomalies associated with a poor neurodevelopmental outcome.
2. The parents having insufficient English to complete the assessment requirements.
3. Families who identify at recruitment that they are unwilling to return to the hospital for the outcome assessment at 24 months C.A.

There will be no barriers for indigenous parents, single mothers, or same-sex parents. Single mothers will be assisted at recruitment to identify a significant other for partner support strategies.

### Sample size

The sample size needs to be sufficient to provide reliable evidence about whether family functioning is sufficiently impacted by the Baby Triple P for Preterm infants intervention to improve the primary outcome of child behavioural and emotional problems. A clinically important difference in child behavioural and emotional problems is considered to be a standardised effect size of 0.33 on the ITSEA [49]. This is a conservative estimate of the expected effect size observed in past RCTs of Triple P [36]. With a type-1 (alpha) level of 0.05, and 80% power, a total of 140 per group are required for analysis. Based on our experience, the expected retention rate is > 85% so that 330 families (n = 165 in each group) will be recruited to allow for attrition. As babies of multiple births cannot be considered independent, the unit of randomisation is family (or delivery), and the total number of babies recruited will depend on the number of multiple births. A total of ~675 families of preterm infants born at <32 weeks who survive to term equivalent are admitted to RBWH and MMH combined over the proposed 3 year recruitment period. Allowing for the estimated retention rate of 50% of those eligible, the feasibility is high of recruitment of a sufficient sample of at least 330 of the eligible 338 families to be studied at 24 months C.A.

### Consent, randomisation, stratification

At each site the recruitment nurse will assess infants for eligibility and approach their parents as soon as the infant is medically stable (as determined by conferring with one of the attending doctors before approaching the parents). At first approach the research nurse will outline key elements of participation (including the voluntary nature of participation), and give a brief outline of both the risks and benefits and specific research activities of the study. Parents who indicate interest at this stage will be given a ‘recruitment pack’ containing printed material about the key elements of participation and a copy of the parent information and consent form (PICF) to read over. After sufficient time to consider participation the recruitment nurse will make a second approach to the parents. For parents who at second approach indicate willingness to participate the recruitment nurse will then go through the PICF in detail with the parents and ask if they have any further questions. Parents who agree to participation will then either sign and return a written consent form or give consent by clicking the “I agree” box at the start of completing the online version of baseline questionnaires.

Once baseline assessment has been completed by the parents the family will then proceed to random allocation to either the Intervention group (Baby Triple P for Preterm infants) or the Care as Usual (CAU) group. Randomisation will occur from concealed envelopes opened in front of the participants by non-study personnel. Treatment allocation will be recorded on a piece of folded paper inside each envelope in random order (the allocation sequence will be comprised of computer-generated random numbers in a block design) with green paper indicating Intervention group and yellow paper indicating the Care as Usual group. Envelopes for randomisation will be stratified for risk of brain injury on routine cranial ultrasound into (i) normal (NAD) or IVH grade I or IVH grade II (ii) IVH grade III or IVH grade IV or Periventricular Leukomalacia (PVL). Multiple births will be assigned to the same group (as the unit of randomisation is the family).

### Blinding

Due to the design of the study participants and intervention delivery facilitators will be informed of group allocation. Therapists conducting the BSID III assessment at 2 years C.A, and coders of the video/audio recorded observations will be masked to group allocation.

### Study treatments

*Content of Baby Triple P for Preterm infants:*

*Hospital based content:*

4 × 2 hr group sessions structured in incremental anticipatory guidance style will guide parents to:

- Identify personal stressors and enhance coping skills (e.g. awareness of common ‘early parent traps’, enhancing social support, relaxation skills, coping strategies)
- Knowledge of effective partner support strategies (e.g. communication skills, awareness of common partner traps, maintaining relationship happiness, negotiating a fair division of labour). Note that single mothers will identify a significant other to use these strategies with at recruitment.
- Recognise their baby’s needs (e.g. feeding/sleep, emotional needs, attention needs)
- Modify environmental influences to facilitate the development of well-organized behaviour
- Teach baby new skills (e.g. engaging activities, giving attention, praise)
- Develop a positive relationship (e.g. promoting alert state, robust sleep, addressing emotional needs)
- Deal with infant behaviours (encouraging contentment, establishing limits, using diversion).
- Deal with crying (why, how much, settling strategies, what to do when crying persists).

*Community based content:*

- 4 × 30 min telephone consultations soon after transfer from hospital with the Baby Triple P for Preterm infants hospital facilitator to allow for tailoring of Baby Triple P for Preterm infants content to individual family needs.
- Ongoing receipt of Triple P tip-sheets plus telephone support as required. Directly linked to local Triple P services already available in the community where possible.
- Fortnightly text messages reiterating program content.

Overall, 4 modules will be delivered as 2 hour group sessions in each hospital by psychology graduates and/or clinical nurses who have completed Triple P facilitator training. A parent workbook accompanies the program and is given to parents for use with exercises and as a reference. Parents complete homework tasks between sessions to consolidate learning. A flexible approach to delivery format is essential. If group sessions are not possible then individual sessions will be provided. Any family discharged from hospital prior to session completion will be provided with a DVD of missed modules to be watched at home followed by a telephone consultation with their facilitator. After hospital discharge and completion of the first four modules, 4×30-min telephone consultations are conducted with the family in their community by the trained Triple P facilitator who conducted the hospital-based sessions. The telephone sessions provide additional support to parents as they put into practice what has been learnt and the facilitator will assist parents in setting and reviewing parenting goals. Upon completion of all 8 sessions participants will be provided with contact details of their nearest community-based Triple P support location and encouraged over a 2 year period to access these services including seminars, groups and enhanced individual support if required. In addition we will provide intervention participants with ongoing intervention maintenance in the form of Triple P tip-sheets every 3 months (providing developmentally appropriate advice on parenting infants) with ongoing phone support if required, and brief fortnightly text messages reiterating program content.

### Care as Usual (control condition)

Infants randomly assigned to the ‘Usual Care’ group will receive standard follow-up after discharge which does not involve a structured preterm parenting program. Exposure to any structured parenting programs will be reviewed by questionnaire at 6 weeks, 12 months and 24 months C.A. by parental recall.

### Monitoring the intervention

Session checklists will be used to monitor the content delivered. In addition, all Baby Triple P for Preterm infants group sessions and telephone consultations will be video/audio recorded to allow independent protocol adherence checks.

### Concurrent interventions

Both groups may be referred for or receive other concurrent therapies during the period of the study (e.g. physiotherapy, speech therapy, community programs). The content and intensity of any concurrent therapy will be documented by parent recall in questionnaires at 12 and 24 months C.A.

### Clinical ethical considerations

Parents who score >12 on the Edinburgh Post-natal Depression Scale at baseline will be referred to the relevant clinician with the parent’s consent. Parents with elevated scores at 6 weeks post baseline, or 12 months C.A, and/or elevated scores at 24 months C.A. on the Depression Anxiety and Stress Scale will be phoned to discuss referral options. If there are concerns of developmental delay, behavioural problems determined or reported at the determined at 24 month assessment, the managing clinician will be notified. Motor function will be classified by the parent and clinician on the Gross Motor Function Classification System at 24 months C.A [50]. These families will remain in the program and will be included in the full analysis.

## Measures

### Sample descriptors and prediction variables

- *Family background questionnaire* (FBQ; assessed at baseline, post intervention, at 12 and 24 months C.A.) Assesses maternal and family factors, socio-economic status, education level and whether it was a planned pregnancy to determine impact on outcome.
- *Medical risk factors* (assessed at baseline) for outcome from the child’s case notes using the standardised Australian and New Zealand Neonatal network data definitions (e.g. GA, birth weight, disease severity, PIVH [50], PVL) [1].
- *The Gross Motor Function Classification system* (GMFCS; assessed at 24 months C.A.) is a valid and reliable way to classify the functional abilities of children with cerebral palsy and physical disability.
- *Social Support Scale* (SSS): will be assessed at baseline, 6 weeks post and 12 months C.A [40]: The SSS is a 4-item measure of the parent’s satisfaction with their social support network. The first two items referred to *formal support* (help that people get from professionals or organisations such as child health nurses or paediatricians) and asked participants to a) list the people they receive formal support from using initials and b) how satisfied they are with the extent of formal support they currently receive (from 1 = *Not at all satisfied* to 5 = *Completely satisfied*). The second two items referred to *informal support* (support that is not paid for and often provided by family, friends and neighbours) and asked participants to a) list the people they receive informal support from using initials and b) how satisfied they are with the extent of informal support they currently receive (from 1 = *Not at all satisfied* to 5 = *Completely satisfied*). Listing people participants receive support from was used as a prompt to think about their support network and these items were not used for data analysis.

### Outcome measures

Assessments will be conducted at baseline, post-intervention (6 weeks) C.A., 12 months (±1 month) C.A. and 24 months (±1 month) C.A. Participants will have the option to complete questionnaires either online or by hardcopy. In the case of hardcopy, questionnaires will be posted to participants (other than at baseline) along with a reply paid envelope for easy return.

### Child outcomes

Primary Outcome Child Behavioural and Emotional Problems:

- *Infant Toddler Social & Emotional Assessment* [49,51] (ITSEA); will be assessed at 12 and 24 months C.A: This is a primary outcome measure of the study (at 24 months C.A.). The ITSEA is a 165-item parent-report questionnaire to assess social-emotional problems/competencies in the domains of behavioural dysregulation; externalising behaviour; internalising behaviour, and competence Items are presented as statements e.g. “Your child is restless and can’t sit still” and rated on a 3-point scale 0 (not true/rarely) 1 (somewhat true/sometimes) 2 (very true/often). This study will utilise only the externalizing, internalizing and dysregulation subscales. The ITSEA has established concurrent validity, strong test-retest reliability (α = .75-.91) and good internal reliability for each subscale (α = .86 for dysregulation, α = .87 for externalising, α = .85 for internalising, and α = .89 for competence) [49,51].
- *Observation of mother-child interaction* over 15-minutes will be video-recorded (at 24 months C.A.) during a series of structured tasks to corroborate parent reported data on child behavioural and emotional problems. For example child behaviours coded for are non-compliance, complaint, aversive demands and positive contact. Observations will be conducted in the hospital and will consist of four timed segments: 3 minutes of free play with toys provided, 5 minutes of a compliance task where the parent instructs the child to work with them on a paper and crayon task involving the parent giving simple instructions to the child; 5 minutes where the parent is made ‘busy’ by completing a questionnaire, and 2 minutes of a second compliance task in which the parent instructs the child to pack up the toys. Steps will be taken to reduce the effects of reactivity by positioning the camera as far away as is reasonable and having the researcher leave the room between segments. Coding of the observations will be done by a researcher and trained research assistants using the Family Observation Schedule (FOS [52];which has demonstrated reliability and discriminant validity [15,48,53,54]. The FOS is a microanalytic coding system in which the presence or absence of particular behaviours of both the child are scored in consecutive 10-s intervals. Where the child has a physical disability the tasks will be modified.
- *Observation of mother-child interaction (6 weeks and 12 months CA).* Participants living within 100 kilometres ‘*as the crow flies’* from Brisbane, will complete a video-recorded mother-infant observation conducted in the mother’s home at 6 weeks and 12 months CA. At 6 weeks CA the researcher will instruct the mother to position herself face-to-face with the infant. The mother will then be instructed to interact the way she normally would with her infant, and to attend to any care the infant may require, for example, feeding. The observation will be recorded for 15-minutes. At 12 months CA the researcher will again instruct the mother to interact the way she normally would with her child, this time through a series of 5 segments: 2-minute interaction with a pop-up toy; a limited physical availability task involving a 3-minute telephone call from the researcher to the mother; 4-minute interaction with a set of blocks; 4-minute interaction with a puzzle; 6-minute interaction with a train set. The mother will be instructed to pack up each toy between segments. The observation will be recorded for 20-minutes. Both observations will be coded using the Emotional Availability Scales (EA Scales) [55] by two independent coders blind to group allocation. The measure assesses the quality of the mother-infant relationship across four caregiver scales (sensitivity, structuring, nonintrusiveness and nonhostility) and two child scales (responsiveness and involvement). The scale has high inter-rater reliability: sensitivity (.89), structuring (.91), nonintrusiveness (.86), nonhostility (.76), responsiveness (.88) and involvement (.87) [56].
- *Baby Behaviour Inventory* (BBI): will be assessed at 6 weeks post and 12 months C.A. The BBI is a 14-item measure of problematic baby behaviour with three scales: an intensity scale, a problem scale and a confidence scale. Each item describes an infant behaviour e.g. “Baby waking more than four times per night”. For the intensity scale parents indicate how often this behaviour occurs with their baby on a 5 point scale 1 (Never) 2 (Seldom) 3 (Sometimes) 4 (Often) 5 (Always). Parents then indicate if they experience the behaviour as a problem (yes/no) (problem scale) and if so then they rate their level of confidence in dealing with the problem on a 5 point scale 1 (Never confident) 2 (Sometimes confident) 3 (Confident) 4 (Very confident) 5 (Extremely confident). The BBI has reasonable internal consistency (α = .84 for overall intensity scale) and test-retest reliability (*r* = .70, .59 and .57 for the intensity, problem and confidence subscales respectively) and demonstrated construct validity [40].

*Child Cognition and Language:*

- *The BSID III* [57] will be administered at 24 months C.A. to assess early cognitive development, language and motor abilities. It takes up to 1.5 hours to administer.
- *The CSBS DP Infant-Toddler Checklist* [58] will be completed by mothers during the ‘parent busy’ segment of the mother-toddler observation conducted at 24 months CA. For use with parents of children from 6 to 24 months of age, the ITC is a broader developmental screen that identifies children with developmental, in particular language and communication delays as well as children with an autism spectrum disorder (ASD). The ITC consists of 24 questions about early social communication behaviour (e.g. "Does your child let you know when he/she needs help or wants an object out of reach?"). Each item is rated on either a 3 or 4-point scale i.e. Not Yet (0), Sometimes [1], Often [2]. Sensitivity of the ITC for identifying developmental delays is 83%.
- *Maternal Infant Responsiveness Instrument* [59] (MIRI); will be assessed at 6 weeks and 12 months CA. The MIRI is a 22-item self-report questionnaire measuring maternal responsiveness to infant cues with high internal consistency (α = 0.87) [60] and face and content validity that has been established using advanced nurse practitioners and maternal child nursing experts [59].
- *Maternal Postnatal Attachment Scale*: (MPAS) will be assessed at 6 weeks and 12 months CA [61]. The MPAS is a 19-item self-report questionnaire measuring mother-to-infant attachment. It has high internal consistency (α = 0.78, 0.79 and 0.78) at four weeks, 4 months, and 8 months respectively, and construct validity that has been established with significant negative correlations with infant temperament and maternal negative affective states [61].
- *Parenting Scale* [62] (*PS)*: assessed at 24 months CA: The PS is a 30-item measure of 3 dysfunctional parenting styles: laxness (α = .83); over-reactivity (α = .82) and verbosity (α = .63). Reliability is strong (α = .84). Each item requires the parent to rate the likelihood of using a particular discipline strategy in response to common child misbehaviours using a 7-point Likert-type scale. Item scores are summed then averaged to give a total score ranging from 1 to 7. The PS is a valid and reliable tool, with good test–retest reliability (r = .84) and strong discriminant validity between parents of clinic/non-clinic children. The PS has been recommended as a tool for measuring parenting skill [62,63].
- *Parenting Scale* (PS-12 M): has been adapted for parents of children aged 12 to 18 months and will be assessed at 12 months CA [64]. A 21-item measure that has been adapted to match the language, behaviour and parent–child interactions appropriate for younger children 12–18 months of age. Each item requires the parent to rate the likelihood of using a particular discipline strategy in response to common challenging behaviours using a 7-point Likert-type scale. Item scores are summed to give a total score. Factor analysis revealed a one factor solution with good test reliability (r = .56, p = .01) and strong convergent validity (r = −.77, p = .006).
- *Maternal Self-Efficacy Scale* (MSES) [44]: will be assessed at 6 weeks post intervention, then at 12 and 24 months CA. The MSES is a 10-item measure of parents’ self-efficacy that has good internal consistency (α = 0.86) and a strong concurrent validity with the PSI Sense of Competency Scale [65]. At 24 months, an adaptation of this scale for toddlers will be used [44].
- *Brief Child Abuse Potential Inventory* [66] (BCAP): will be assessed at 24 months CA. The BCAP is a 33-item measure of the potential for child abuse with strong internal consistency (α = 0.89) and correlation (*r* =0.96).
- *Edinburgh Postnatal Depression Scale* [67] (EPDS): will be assessed at baseline, at 6 weeks post intervention then at 12 months CA. The EPDS is a 10-item screen for postpartum depression with good internal (α = 0.80) and test-retest (α = 0.77) reliability.
- *Depression, Anxiety and Stress Scale-21* [68] will be assessed at 24 months C.A. The DASS-21 is a 21 self-report item questionnaire reflecting the frequency or severity of the participant’s experiences with depression, anxiety and stress over the past week with high internal consistency (α = 0.83, 0.78 and 0.87 for depression, anxiety and stress respectively [69]. High convergent validity has been established between the DASS and other measures of similar constructs: DASS depression scale and the Beck Depression Inventory *(r* = .76), DASS anxiety scale and the Beck Anxiety Scale (*r =* .74*)* and DASS stress scale and the Positive and Negative Affect Schedule (*r* = .74) [70].
- *Relationship Quality Index (RQI* [69]; will be assessed at baseline, at 6 weeks post intervention then at 12 and 24 months CA [69]. The RQI is a 6-item questionnaire of relationship satisfaction with good internal consistency (α = .68-.86), reliability (α = .90) and discriminant validity. The scale consists of 5 specific items regarding relationship health and 1 global item reflecting global overall relationship satisfaction. A higher score corresponds to higher relationship satisfaction. The RQI has good internal consistency (α = .68-.86;) [69] and reliability (α = .90).
- *Frequency and Acceptability of Partner Behaviour* (FAPBI) [71]: will be assessed at baseline, at 6 weeks post intervention, then at 12 months CA. The FAPBI is a 19-item measure of the frequency and acceptability of positive and negative partner behaviours with strong internal consistency (α = .73-.85), correlation (*r* = .43-.58) with partner-reports and strong discriminant validity between couples seeking marital therapy and non-clinic couples.
- *Rapid KPI coding system* [72]: will be assessed at baseline, then at 6 weeks post treatment and 12 months C.A. The KPI will be used to code a video or audio recorded 10-min observation of couple interaction for parents in a relationship, discussing a topic of current conflict. Parents will be provided with a list of possible topics centring on baby care tasks or couple issues. Parents living within a 100 km radius of Brisbane will complete the task either in-home or at the hospital, video-recorded by a researcher. Couples who live beyond 100kms will receive an audio-recorder to complete the task independently, and will post this back using a reply paid envelope. Couple communication is scored in 30s intervals for conflict, validation and verbal affect. The KPI has good internal consistency (positive discussion: κ = .65, validation: κ = .58, invalidation κ = .69, conflict κ = .62, and negative nonverbal behaviour κ = .59 [72], is sensitive to change and has been used to measure changes in couple communication from couple’s therapy [72-74].
- *Parent Problem Checklist – Baby* (PPC-B): will be assessed at 6 weeks post intervention then at 12 months CA [75]. The PPC-B is a 16-item questionnaire which measures conflict between parents over child rearing. The PPC-Baby has demonstrated strong internal consistency (α = .70) and good test-retest reliability (*r* = .90) with a subset of clinic parents who completed the scale again after an 8-week interval [75].
- *Parenting and Family Adjustment Scale* [76]. (PAFAS): will be assessed at 24 months C.A. The PAFAS assesses parenting practices and parent and family adjustment. It consists of a 28-item Parenting Scale encompassing two domains including parenting practices (17 items) and parent–child relationship (11 items) and of a 12-item Family Adjustment scale encompassing three domains including parental emotional maladjustment (5 items), family relationships (4 items) and parental teamwork (3 items). Each item is rated on a 4-point scale from not true of me at all (0) to true of me very much [3]. This study will use only the three items from the parental teamwork domain which has good internal consistency (coefficient H = .85). The PAFAS has satisfactory construct and predictive validity [76].
- *Client Satisfaction Questionnaire* (CSQ): will be administered at 12 and 24 months C.A. for the intervention group only. The CSQ is a 10 item measure of the parent’s satisfaction with the parent training program. It is an adaptation of the Therapy Attitude Inventory (TAI) [77]. The TAI has established reliability, internal consistence and discriminant validity.

### Analyses

Analysis will follow standard principles for randomised controlled trials using 2-group comparisons performed using all subjects for whom outcome data are available, on an intention-to-treat basis. The experimental unit is a family. The effect of clustering for children in multiple births will be partitioned from the experimental error, which will be estimated from the between family variability. Up to 50% of eligible families may not consent or alternatively, be back-transferred to regional hospitals before an approach can be made, and a further 15% may be lost to follow-up at 2 years. Consequential threats to external and internal validity will be checked using baseline and descriptive information for eligible families. Imputation techniques will be used to avoid bias which may be a consequence of non-ignorable missing data during follow-up. All data analyses were performed using STATA 10.0 (Statacorp 2007).

The primary endpoint at 24 months C.A. is the child behavioural and emotional problems as measured on the ITSEA and the mother-toddler observation. This comparison will be between treatment groups using general linear models, with terms included for stratification and important confounding variables, such as the extent of brain injury and socio-demographic variables. Secondary analyses will use similar methods to compare the outcomes between groups for the additional outcomes (cognition, language etc.). For dichotomous outcomes, comparison will be by chi-square tests and multiple logistic regression. Where continuous data exhibit substantial skewness not overcome by transformation, non-parametric methods (Mann–Whitney *U* test) will be used for simple comparisons. Further analysis will be performed using latent growth modelling (LGM) [78,79] as it provides simultaneous analysis of time points and can incorporate latent measures corrected for measurement error [80]. The added-growth LGM used in evaluating the efficacy of Baby Triple P for Preterm infants tests the extent to which the intervention alters the trajectory of the target behaviour [79]. The models may incorporate main effects of time-independent and dependent covariates (e.g. control variables) on trajectories, as well as interactions of such measures with intervention effects [78,79]. The models express the expected change in dependent variables as a function of exposure to the intervention (e.g. Baby Triple P for preterm infants versus Control).

## Discussion

This paper outlines the background and design for an RCT to determine whether Baby Triple P for Preterm infants compared to Care as Usual optimises child outcomes including behavioural and emotional adjustment, and cognitive and language development at 24 months C.A. in infants born very preterm. The lack of sustained treatment effects for existing interventions suggests that an intervention that focuses on sustained environmental enrichment through enhanced parenting practices may be beneficial. Baby Triple P for Preterm infants is designed to enhance the knowledge, skills and confidence of parents of preterm infants. It has the potential to make a significant contribution to positive family relationships, good infant development and to the reduction of psychological adjustment difficulties and improved behavioural, language and cognitive development leading to improved educational outcomes all of which are important in functional community contribution in later years.

### Ethics approval

Ethical permission to conduct the study has been obtained from Qld Children’s Health Services, Human Research Ethics Committee (HREC/08/QRCH/114) and the University of Queensland (2008002268).

### Data sharing statement

TE, MH, JA will use data from this study to contribute to their PhD theses.

## Abbreviations

AU: Care as Usual
RCT: Randomised Controlled Trial
IVH: Intraventricular haemorrhage
PVL: Periventricular leukomalacia
BSID III: Bayley Scales of Infant and Toddler Development
CA: Corrected age
CP: Cerebral palsy
GA: Gestational age
PMA: Post menstrual age
